# The Immediate Early Response of Lens Epithelial Cells to Lens Injury

**DOI:** 10.3390/cells11213456

**Published:** 2022-11-01

**Authors:** Samuel G. Novo, Adam P. Faranda, Mahbubul H. Shihan, Yan Wang, Ananya Garg, Melinda K. Duncan

**Affiliations:** Department of Biological Sciences, University of Delaware, Newark, DE 19716, USA

**Keywords:** lens, cataract surgery, wound healing, immediate early genes, inflammation

## Abstract

Cataracts are treated by lens fiber cell removal followed by intraocular lens (IOL) implantation into the lens capsule. While effective, this procedure leaves behind numerous lens epithelial cells (LECs) which undergo a wound healing response that frequently leads to posterior capsular opacification (PCO). In order to elucidate the acute response of LECs to lens fiber cell removal which models cataract surgery (post cataract surgery, PCS), RNA-seq was conducted on LECs derived from wild type mice at 0 and 6 h PCS. This analysis found that LECs upregulate the expression of numerous proinflammatory cytokines and profibrotic regulators by 6 h PCS suggesting rapid priming of pathways leading to inflammation and fibrosis PCS. LECs also highly upregulate the expression of numerous immediate early transcription factors (IETFs) by 6 h PCS and immunolocalization found elevated levels of these proteins by 3 h PCS, and this was preceded by the phosphorylation of ERK1/2 in injured LECs. Egr1 and FosB were among the highest expressed of these factors and qRT-PCR revealed that they also upregulate in explanted mouse lens epithelia suggesting potential roles in the LEC injury response. Analysis of lenses lacking either Egr1 or FosB revealed that both genes may regulate a portion of the acute LEC injury response, although neither gene was essential for expression of either proinflammatory or fibrotic markers at later times PCS suggesting that IETFs may work in concert to mediate the LEC injury response following cataract surgery.

## 1. Introduction

The lens is a transparent tissue that refracts light onto the retina and is thus critical for high resolution vision [1]. Cataract, the clouding of the ocular lens due to age, metabolic dysfunction, genetic abnormalities, or ocular injury, was historically the predominate cause of human blindness [2]. However, over the past 50 years, the global burden of cataract has been greatly reduced due to the development of cost effective extracapsular/phacoemulsification cataract extraction methods coupled with the implantation of artificial intraocular lenses which restore vision [3].

While modern cataract surgery has revolutionized the practice of ophthalmology, like all surgeries, it results in elevated inflammation at the wound site [4,5,6] leading to post-surgical pain and discomfort. As ocular inflammation can also trigger retinal edema, retinal detachment, and exacerbate uveitis [6,7,8,9,10] as well as fibrotic conditions [11,12], it is typically aggressively treated with a combination of steroids and NSAIDs either via eye drops [13,14] or “drop-less” methods [15] which instill anti-inflammatories into the eye at the time of surgery. While these drugs are highly effective, post-surgical inflammation still does occur, and is clinically observed as “flare” [16] defined by increases in the protein concentration of the aqueous humor and the influx of inflammatory cells into the anterior segment of the eye by 20–24 h post cataract surgery (PCS) [17,18].

Concomitant with inflammation, lens epithelial cells (LECs) left behind after cataract surgery undergo a wound healing response characterized by their increased proliferation and migration which occurs simultaneously with their phenotypic conversion into either myofibroblasts [19] or aberrant lens fiber cells [20,21]. Early in the development of extracapsular cataract surgery methods, these migratory lens–derived cells (LCs) would rapidly populate the posterior lens capsule, leading to the development of posterior capsular opacification (PCO) within weeks or months following cataract surgery. However, advanced IOL designs now physically sequester LCs to the capsular bag periphery, preventing their migration into the optical axis [11], which greatly reduced the incidence of PCO within a year following surgery. However, longer term, PCO is still of clinical concern as lens derived myofibroblasts persist years or even decades following cataract surgery [22], and can escape from the capsular bag periphery leading to PCO development at extended times PCS [23,24].

While the pathogenesis of ocular inflammation PCS has been attributed to mechanical disruption of the blood/aqueous barrier during surgery [25,26], the underlying molecular mechanisms driving this have not been well characterized. However, it has long been recognized that poor control of inflammation is associated with increased risks of retinal damage and aggressive PCO development PCS highlighting the importance of studying its etiology [7]. We recently discovered that the LECs remaining behind after lens fiber cell removal in both mouse [18,27,28] and human (manuscript in preparation) models of cataract surgery massively remodel their transcriptome by 24 h PCS to express high levels of both pro-inflammatory cytokines and fibrotic marker mRNAs. While our prior study found that LECs express elevated protein levels of some inflammatory cytokines by 3–6 h PCS [18], neither the extent of this initial pro-inflammatory response nor potential mechanisms regulating it were known.

Here, we used RNAseq to globally profile the transcriptional changes that mouse LECs undergo by six hours after fiber cell removal in a mouse model of cataract surgery in order to characterize the acute response of these cells to surgery and tested the function of two potential regulators of this initial response.

## 2. Methods

### 2.1. Animals

All procedures using mice comply with the Association for Research in Vision and Ophthalmology on the Use of Animals in Vision and Ophthalmologic Research and were approved by the University of Delaware Institutional Animal Care and Use Committee under protocol #1039-2021-1. Animals were bred and maintained in ventilated caging at the University of Delaware animal facility under a 14/10 h light-dark cycle and received food ad libitum.

Mice harboring a germline deletion of the Egr1 (Egr1KO) gene were obtained from the Jackson Laboratory ((JAX stock #012924; B6N; 129-*Egr1^tm1Jmi^*/J) [29], where they were deposited by Dr. Jeffery Milbrandt, Washington University School of Medicine, St. Louis. These mice were genotyped for lack of an intact Egr1 gene via PCR analysis utilizing the following primers: Common forward: 5′-GGG-CAC-AGG-GGA-TGG-GAA-TG-3′, Wildtype reverse: 5′-AAC-CGG-CCC-AGC-AAG-ACA-CC-3′, Mutant reverse:5′-CTC-GTG-CTT-TAC-GGT-ATC-GC-3′.

Mice harboring a FosB allele where exon 2 and 3 are flanked by lox *p* sites (Fosb^tm1.1Nes^) [30]. were obtained from Drs. Alfred Robinson (Michigan State University) and Eric Nestler (Icahn School of Medicine at Mount Sinai). MLR10 cre mice (Tg(Cryaa-cre)10Mlr) binding site modified alpha-A crystallin promoter [31] were obtained on an FVB/N background from Dr. Michael Robinson (Miami University, Oxford, OH, USA) and backcrossed to C57Bl/6 mice for over 10 generations at the University of Delaware. Mice lacking the FosB gene from the lens (FosBcKO mice) were generated by mating Fosb^tm1.1Nes^ mice with Tg(Cryaa-cre)10Mlr mice. Mice were genotyped for the presence of the floxed FosB allele, and MLR10cre transgene via PCR utilizing the following primers: FosB flox forward: 5′-GCT-GAA-GGA-GAT-GGG-TAA-CAG-3′ and reverse: 5′-AAG-CCT-GGT-ATG-GTG-A-3′: MLR10cre forward: 5′-CCT-GTT-TTG-CAC-GTT-CAC-CG-3′ and reverse: 5′-ATG-CTT-CTG-TCC-GTT-TGC-CG-3′. Successful deletion of exon 2 and 3 of the FosB gene was confirmed by PCR using genomic DNA isolated from adult mouse lenses utilizing the following primers: forward: 5′-TTC-CCT-TCC-TAT-TTG-TAG-AGC-GTA-G-3′ and reverse: 5′-TGC-TAC-TTG-TGC-CTC-GGT-TTC-C-3′.

### 2.2. Morphological Analysis

Histological analysis was conducted by isolating eyes from euthanized mice and immediately fixing them in Davidson’s Fixative Solution (Electron Microscopy Sciences, Hatfield, PA, USA). The fixed eyes were sent to HistoWiz (Brooklyn, NY, USA), where they were sectioned and stained with hematoxylin and eosin (H&E). The H&E stained slides were then photographed on a Zeiss Axio Observer epifluorescent microscope (Carl Zeiss Inc., Gottingen, Germany).

Lens clarity was assessed by viewing isolated lenses using darkfield optics. Lens optical properties were assessed following previously described methods [32,33] by placing the lenses on a 200-mesh electron microscopy grid. Images were collected with a Zeiss Stemi SV microscope fitted with a darkfield base (Carl Zeiss Inc., Gottingen, Germany).

Lens measurements were conducted by taking images of the reflection of the lens through a 45° mirror (face = 5 mm × 5 mm) (Edmund Optics, Barrington, NJ, USA) as previously described [34] in a plastic box with 1XPBS. The mirror was positioned at a constant distance from the lens and an in-focus image of the mirror edge (length = 5 mm) was also collected for distance calibration. Image measurements were calculated by Fiji ImageJ v52 [35] based on the distance calibration.

### 2.3. Mouse Cataract Surgery Model

Lens fiber cells were surgically removed from living 3–5 months old mice of both sexes to simulate the extracapsular lens extraction surgery typically performed to treat human cataract [36,37]. Notably, this surgery differs from human cataract surgery in that an anterior capsulotomy is not performed and the lens fiber cell mass is removed as a single unit. Generally, no additional aspiration of lens cortical material is needed for complete fiber cell removal in this method. Samples were collected from euthanized animals at various times post fiber cell removal including 0, 1, 3, 6, 12, 24, 48, 72, and/or 120 h post surgery (post cataract surgery, PCS).

### 2.4. Next Generation RNA Sequencing and Bioinformatic Analysis

Lens capsular bags with the attached lens cells (LCs) were dissected at either 0 or 6 h PCS. Samples from five independent animals were pooled to create a single biological replicate, then flash frozen on dry ice as previously described [27,28].

Total RNA was extracted from each biological replicate using the RNeasy Mini Kit from Qiagen (Cat No./ID: 74104). RNA libraries were prepared using the SMARTer^®^ Stranded Total RNA-Seq Kit-Pico Input Mammalian (Takara Bio USA, Inc., Mountain View, CA, USA) and sequenced by DNA Link, USA (1000 S Hope St. unit 521 Los Angeles, CA 90015, USA) on a Novaseq 6000 (Los Angeles, CA, USA). Read pairs (101 nucleotides long) were aligned to the Ensembl primary assembly of the mouse GRCm38 genome [38] using the default parameters of Hisat2 [39]. Using the HTseq-Count in union mode [40], read pairs aligned to the genomic features annotated in an Ensembl Mouse GTF file were quantified as gene level counts. The Ensembl version 100 GTF file was used for the initial analysis, and the Ensembl version 104 GTF File was used for later analyses. Following the merging of overlapping exons, length normalized abundance estimates, Fragments per Kilobase-Million (FPKM), were calculated from gene level counts using the total length of all known exons for a given gene.

Samples were partitioned for Trimmed Median of Means (TMM) scaling [41,42] and differential expression analyses were performed based on the objective of a particular contrast.

The edgeR statistical package (version 3.30.3) was used to estimate the magnitude and statistical significance of differential gene expression, with robust dispersion estimates [41,42]. For the initial analysis, edgeR’s “Quasi-Likelihood F-Test (QLF-Test) was used for differential expression analysis. It was later determined that in certain cases, the QLF-Test may be overly conservative for data derived from this experiment, thus edgeR’s “exact test” was used for the subsequent analysis. As significant effort had been applied to the interpretation of the initial study results, and DEG detected by the QLF-Test are usually also captured by the exact test, reanalysis of the initial study was considered unnecessary. Genes with at least 10 mapped reads in at least three samples were considered to have “detectable” levels of expression. Genes failing “detectable” criteria were removed using edgeR’s “filterByExpr” function [43], prior to running statistical tests. Biologically significant differentially expressed genes (DEGs) were characterized as genes exhibiting a statistically significant difference in expression using edgeR’s default method to adjust for False Discovery Rate (FDR ≤ 0.05); a difference in expression level greater than 2 FPKM between PCS conditions, Fold Change (FC) greater than 2 in either the positive or negative direction and expressed at a level greater than 2 FPKM as previously used [44].

### 2.5. Pathway Analyses

iPathwayGuide (Advaita Bioinformatics, Plymouth, MI, USA) pathway analysis was performed on all statistically significant DEGs defined as those exhibiting a fold change ≥ |2| and FDR ≤ 0.05. iPathwayGuide uses Impact Analysis, which considers the directed interactions of DEGs within a pathway (as defined by the Kyoto Encyclopedia of Genes and Genomes, KEGG [45], Release 96.0 + 11–21, Nov 20) and if more pathway participants are observed in the DEG list than would be estimated by chance [46,47,48]. Gene ontology comparisons were made against the 14 October 2020 release of the Gene Ontology Consortium database [49].

### 2.6. Lens Dissections, Culture and RNA Extractions

Mouse lenses were dissected from the eye after mouse sacrifice using sterile forceps. The lens capsule was removed from the lens then suspension cultured in Medium 199 (ThermoFisher Scientific, Waltham, MA, USA) with 1% penicillin-streptomycin for 6 h or immediately placed into 600 µL of Trizol (two lens capsules/biological replicate; ThermoFisher Scientific, Waltham, MA, USA) and stored at −20 °C. After 6 h of culture, the explants were also placed in Trizol (2 capsules per biological replicate) and stored at −20 °C. Crude RNA was isolated following manufacturer methods and was further purified using the RNA Clean & Concentrator^TM^-5 kit (ZYMO Research, Irvine, CA, USA). Isolated RNA was stored in −80 °C until use.

### 2.7. cDNA Synthesis and Quantitative RT-PCR

cDNA was synthesized from lens cell RNA using the iScript cDNA Synthesis Kit (Bio-Rad, Hercules, CA and used for real-time quantitative PCR (qRT-PCR) conducted using the Power SYBR Green kit (Invitrogen Life Technology, Carlsbad, CA, USA) using the gene-specific primers shown in Supplementary Table S1. Three biological replicates were analyzed with 2 technical replicates each for each experimental condition and fold-change calculated using the ΔΔCT-method with GAPDH as the housekeeping gene. Statistical significance was determined using a Student’s two sample *t*-test.

### 2.8. Immunofluorescence and Confocal Imaging

Quantitative immunofluorescence was conducted to determine protein level expression in ocular tissue as previously described [50,51]. Briefly, eyes were placed in Optimum Cutting Temperature (OCT) Media (Tissue Tek, Torrance, CA, USA) following harvest and stored at −80 °C. The frozen blocks were then sectioned at 16µm using the Leica CM3050 cryostat (Leica Microsystems, Buffalo Grove, IL, USA) and placed on Color Frost plus microscope slides (Fisher Scientific, Hampton, NH, USA). Slides were fixed under conditions chosen to best optimize staining for the antibody of interest (Supplementary Table S2). Following fixation, blocking buffer optimized for each antibody (100 µL) was applied to each slide for 1 h at room temperature in a humid chamber (see Supplementary Table S2). Immediately afterwards, the primary antibody diluted in blocking buffer is applied and incubated for either 1 h at room temperature or overnight in 4 °C depending on the antibody (Supplementary Table S2). After primary antibody incubation, slides were washed three times in 1XPBS for 5 min, then the species appropriate Alexa Fluor 488/568 (ThermoFisher Scientific, Waltham, MA, USA) conjugated secondary antibody (if needed) is used at a 1:200 dilution in blocking solution which includes a 1:2000 dilution of Draq-5 (ThermoFisher Scientific, Waltham, MA, USA) to stain DNA and incubated for 1 h at room temperature. After washing, samples were wet mounted in a glycerol based antifade and cover slipped prior to imaging.

During confocal imaging, the negative control was used to measure minimum laser threshold levels to subtract from other experimental images [50,51], in order, to set a baseline, which removes low levels of non-specific binding caused by the secondary antibody. Each immunofluorescence staining experiment was performed with a minimum of three biological replicates. Imaging of the lens capsule and associated lens cells was performed on either a Zeiss LSM780 or Zeiss LSM880 confocal microscope (Carl Zeiss Inc., Gottingen, Germany). The confocal imaging of all slides in the same experiment were imaged the same day using the same imaging parameters to allow direct comparison of experimental conditions and reduce variability [51]. For some figures, brightness and contrast were adjusted post confocal acquisition for optimum viewing on various platforms, but in all cases identical adjustments were on images to be directly compared.

### 2.9. ImageJ Quantification and Statistical Analysis

Immunofluorescence intensities were quantified on a minimum of three independent biological replicates as previously described (Shihan et al., 2021a). All statistics were performed using either a Student’s *t*-test (correct for multiple comparisons using the Holm-Šídák method) or one-way ANOVA with Tukey’s post hoc test. Data are presented as mean ± standard error (SE) with statistical differences considered significant when *p* ≤ 0.05.

## 3. Results

Lens epithelial cells of both mice and humans drastically upregulate the expression of numerous inflammatory mediators and fibrotic marker genes by 24 h post cataract surgery (PCS) [18,52]. However, the mechanisms by which lens fiber cell removal drives this transcriptomic shift were unknown. As the earliest change we have observed in lens-derived cells (LCs) post fiber cell removal is the initial upregulation of inflammatory mediators at 6 h PCS [18], we conducted an unbiased RNAseq analysis on wild type mice at 6 h PCS to elucidate potential mechanisms regulating this process. The raw and processed data for this analysis is deposited in the Gene expression omnibus database under accession number GSE206563.

### 3.1. Lens Epithelial Cells Upregulate the Expression of Numerous Pro-Inflammatory Cytokines by 6 h PCS, but Do Not Yet Upregulate the Expression of Fibrotic Markers

**Figure 1:**
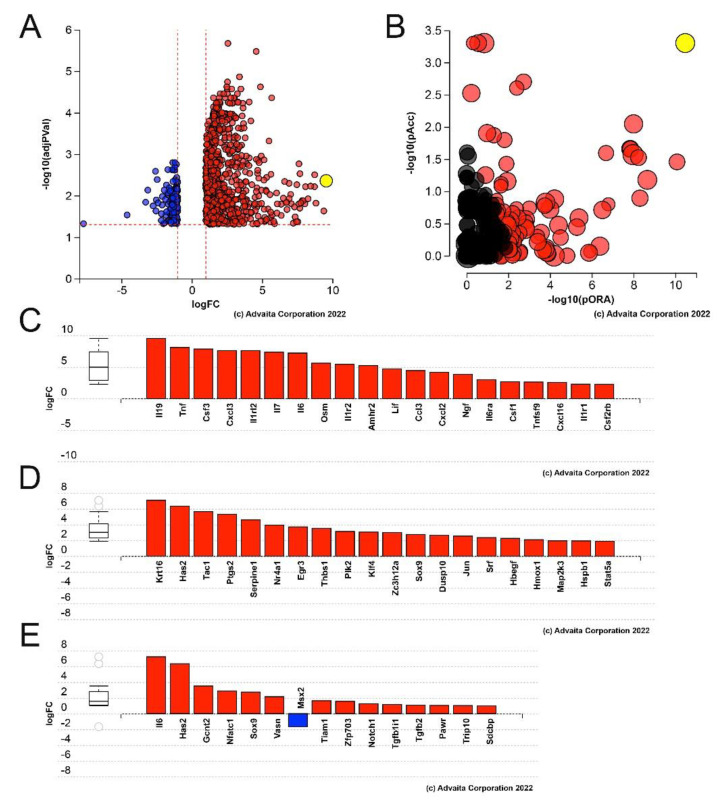
Advaita iPathway analysis of genes differentially expressed at 6 h PCS in LECs. (**A**) Volcano plot of genes (represented by either a blue dot for negative fold change or red dot for positive fold change) whose expression was statistically different in 6 h PCS LECs. Yellow dot represents Il19. (**B**) Impact analysis of the 6 h PCS DEGs predicting that the KEGG pathway “cytokine-cytokine receptor interaction” (*p*-value of 1.120 × 10^−8^) (yellow dot) is the most significantly impacted pathway. While other impacted KEGG pathways are represented by the other dots with black dots being non-significant KEGG pathways and red dots being significant KEGG pathways. (**C**) Bar graph showing which cytokine-cytokine receptor interaction genes are differentially expressed in LECs at 6 h PCS. (**D**) Bar graph showing the “epithelial cell migration” (*p*-value = 9.600 × 10^−5^) genes differentially expressed in 6 h PCS LECs. (**E**) Bar graph showing the DEGs implicated in the “epithelial to mesenchymal transition” (*p*-value = 0.003) pathway.

RNAseq revealed that the normal adult mouse epithelium expresses mRNAs encoded by 17,981 genes while 913 genes were differentially expressed at 6 h PCS by at least two fold at a false discovery rate (FDR) corrected *p* value ≤ 0.05. Filtering these differentially expressed genes (DEGs) for their likelihood to effect cellular biology (“biologically significant” genes exhibit a minimum expression level of 2 FPKM in either condition and at least a 2 Fragments per Kilobase-Million (FPKM) absolute change in expression level [44]), as previously described, revealed 615 DEGs with 547 DEGs being upregulated and 68 DEGs being downregulated (Figure 1A, see Supplementary Table S3).

Advaita iPathway Guide analysis of the DEGs detected in lens epithelial cells (LECs) at 6 h PCS revealed that, like at 24 h PCS [18], the “cytokine-cytokine receptor interaction” pathway was the most impacted with a *p*-value of 1.120 × 10^−8^ (Figure 1B). Of the 615 biologically significant DEGs, the top most upregulated gene at 6 h PCS was Il19, which is implicated in the inflammatory response [53] (Figure 1A, Table 1) while the mRNA for Ptgs2 which encodes the cyclooxygenase Cox2 was upregulated 37 fold and Csf3 was upregulated 233 fold (Table 1) consistent with our prior observation that both Cox2 and Csf3 were upregulated at the protein level in LECs by 6 h PCS [18].

In addition, the DEGs detected at 6 h PCS were significantly enriched in those mapping to pathways necessary for posterior capsular opacification (PCO) pathogenesis including those involved in “epithelial cell migration” (*p*-value = 9600 × 10^−5^) and “epithelial to mesenchymal transition” (*p*-value = 0.003) (Figure 1D,E). Despite this, fibrotic marker mRNAs commonly associated with fibrotic PCO pathogenesis are not significantly upregulated in LECs by 6 h PCS (Table 2) which suggests that LECs induce inflammatory responses prior to fibrotic responses after lens injury.

**Figure 2:**
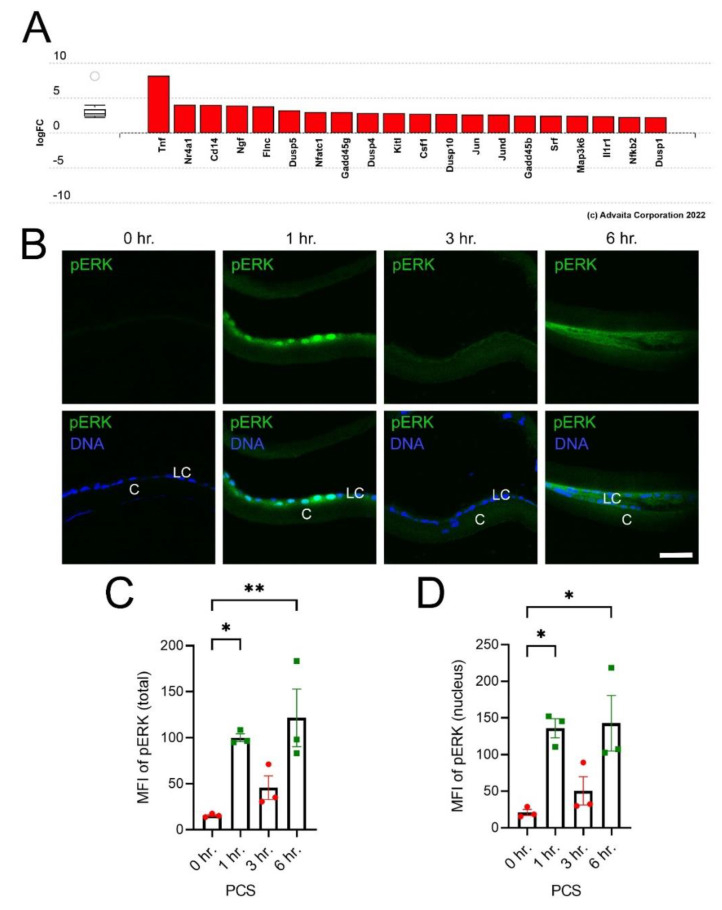
MAPK signaling is acutely activated in LECs after lens injury (**A**) Bar graph of Advaita iPathway analysis of genes differentially expressed in LECs at 6 h PCS associated with MAPK signaling. (**B**) Immunostaining of pERK1/2 (green) in LECs isolated either immediately after lens fiber cell removal (0 h) or 1, 3 or 6 h later. Blue represents DNA. Scale bar = 35 μm (**C**) Quantitiation of total pERK1/2 levels in LECs obtained from three independent biological replicates (**D**) Quantitation of nuclear pERK1/2 levels in LECs obtained from three biological replicates. * represents *p* ≤ 0.05; ** represents *p* ≤ 0.01. The red dots and green squares in C & D both represent the individual biological replicate values for their corresponding bar in the bar graph.

While these data revealed that numerous genes were rapidly upregulated PCS, the mechanisms driving this upregulation were unclear. Notably, Advaita iPathway guide Impact analysis of the DEGs in LECs 6 h after lens fiber cell removal revealed that genes involved in Mitogen activated kinase (MAPK) pathways were enriched in this gene set (Figure 2A; *p*-value = 1.165 × 10^−6^). Immunostaining of LECs for phosphorylated ERK1/2 (pERK1/2) revealed that nuclear pERK1/2 levels were sharply elevated at 1 h PCS, decrease by 3 h PCS, then begin to increase again to variable extents by 6 h PCS (Figure 2B–D).

### 3.2. Many Immediate Early Transcription Factors Are Upregulated in LECs by 6 h PCS

The changes in the LEC transcriptome at 6 h PCS revealed that LECs rapidly induce the expression of many genes after lens fiber cell removal, however, it was less clear how these changes in RNA levels are regulated. Interestingly, FosB, a member of AP1 family of transcription factors, was one of the most induced and abundantly expressed upregulated genes (130 fold change; FPKM = 198) in LECs at 6 h PCS (Table 1). FosB is also recognized to be an immediate early transcription factor (IETF) as its expression is often rapidly induced by cell signaling cascades such as MAPK [54,55]. Notably, comparison between the list of genes differentially expressed in LECs at 0 versus 6 h PCS with a list of IETFs identified in other studies [56,57], revealed that the expression of 27 different IETFs are induced by LECs at 6 h after lens fiber cell removal (Table 3).

### 3.3. IETF MRNA and Protein Levels Acutely Upregulate in LECs at 6 h PCS then Rapidly Fall

**Figure 3:**
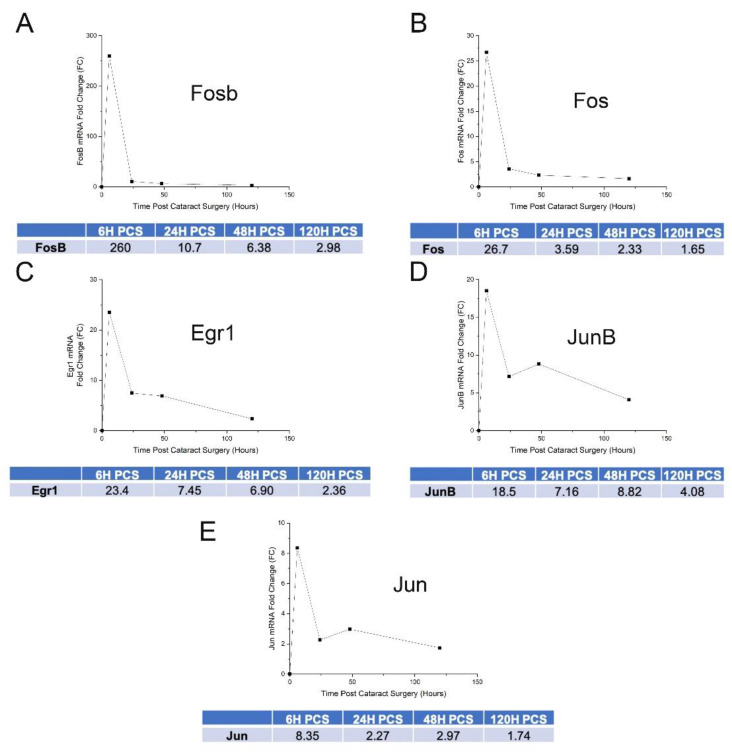
Expression dynamics of select IETFs as revealed by bulk RNAseq expression profiling performed between 0–120 h PCS. (**A**) mRNA expression time course for FosB. (**B**) mRNA expression time course for Fos (cFos). (**C**) mRNA expression time course for Egr1. (**D**) mRNA expression time course for JunB. (**E**) mRNA expression time course for Jun (cJun). The Y axis represents fold expression level in comparison to levels observed in naïve LECs.

**Figure 4:**
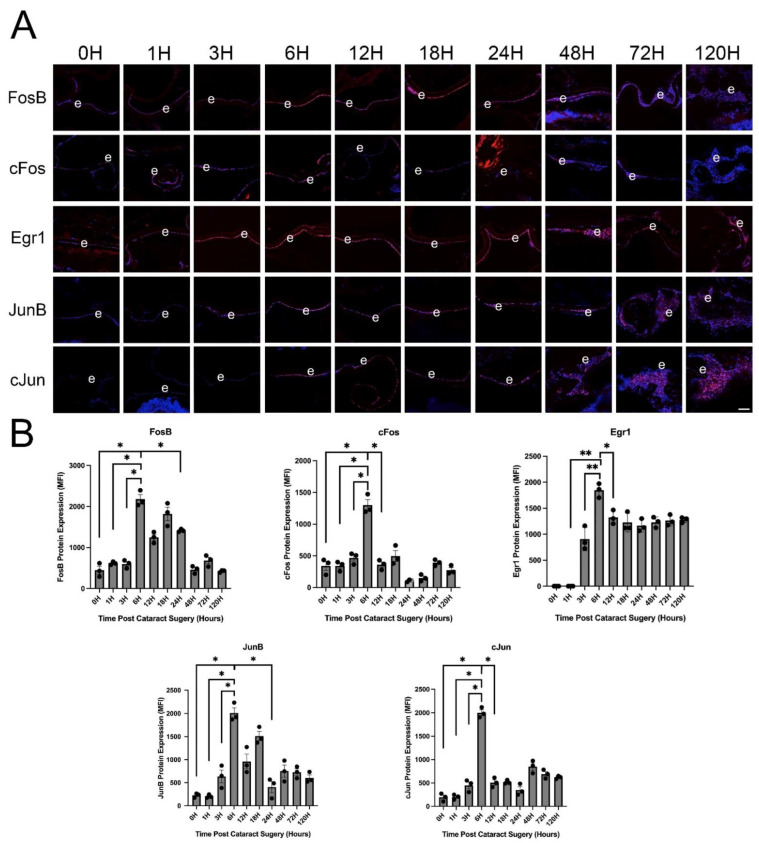
Protein expression of select IETFs in injured mouse lens epithelial cells at various times post lens fiber cell removal. (**A**) Immunolocalization of each studied IETF during the first 120 h (5 days) PCS. Red represents the IETF of interest, Blue represents DNA/cell nuclei. Scale bar = 70 μm. (**B**) Bar graphs representing quantification of the data shown in panel A. FosB * *p*-value ≤ 0.0001; cFos * *p*-value ≤ 0.0001; Egr1 * *p*-value ≤ 0.002 ** *p*-value ≤ 0.0001; JunB * *p*-value ≤ 0.0001; cJun * *p*-value ≤ 0.0001. The black dots in B represent the individual biological replicates for each bar in the bar graph.

**Figure 5:**
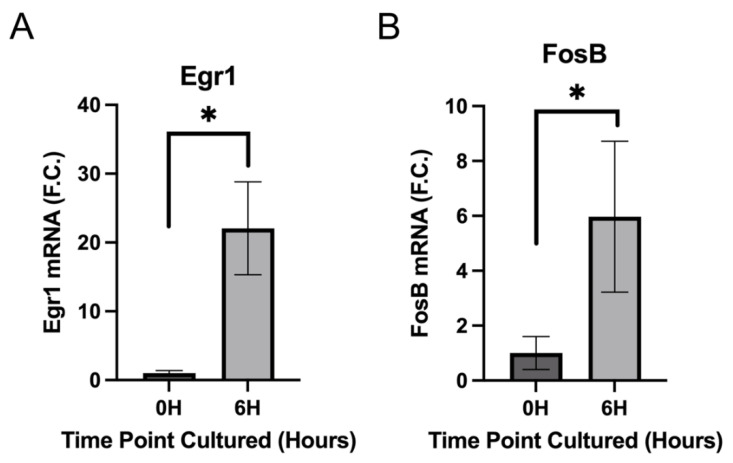
Investigation of the effect of 6 h ex vivo culture on Egr1 and FosB levels in LECs by qRT-PCR (**A**) Egr1 mRNA levels significantly upregulate in LECs after six hours of ex vivo culture (* *p*-value ≤ 0.01) (**B**) FosB mRNA levels signficantly upregulate in LECs after six hours of ex vivo culture (* *p*-value ≤ 0.025) FC-fold change from RNA levels obtained from LECs immediately after isolation.

We have compiled a database of global gene expression changes occurring in LECs between 0–120 h PCS as measured by bulk RNAseq (lens injury response time series/LIRTS, unpublished) and used this to discover the dynamics of IETF expression in LECs from 0–120 h PCS. This revealed that the mRNA levels of these genes are typically highly induced at 6 h PCS, then sharply downregulate by 24 h PCS (Figure 3). As IETFs have the potential to be mediating the LEC wound healing response, we then validated whether a select set of the IETFs whose mRNA levels upregulate in injured mouse lenses in vivo are also upregulated at the protein level. This analysis revealed that the protein levels of FosB, Egr1, Fos (cFos), JunB and Jun (cJun) all upregulate robustly and significantly in LECs by 6 h PCS, and then begin to downregulate over the next 5 days (Figure 4). Next, we attempted to understand whether this rapid induction of IETF expression in LECs PCS was driven by a cell autonomous LEC response or was triggered by something in the ocular environment. Lenses were dissected, and the lens capsule with attached LECs was either immediately frozen or placed into culture in serum free media for six hours. qRT-PCR analysis of these samples revealed that both FosB and Egr1 mRNA levels upregulate in LECs after 6 h of culture (Figure 5) demonstrating that IETF induction by LECs following injury can occur in the absence of signals from other ocular structures.

### 3.4. Removal of the Gene Encoding the IETF Egr1 from the Lens Only Has a Minor Impact on Lens Phenotype

While IETF expression acutely upregulates in LECs at both the RNA and protein levels PCS, their role in the lens injury response was unknown. Notably, Egr1, the most abundantly expressed IETF at the mRNA level (Table 3) which also upregulates at the protein level at 6 h PCS (Figure 4), is a negative regulator of myopia [58] whose expression upregulates in the lens following selenite stress [59] as well as Hsf4 [60] and β1-integrin gene deletion [61]. While the functional importance of Egr1 in the wild type lens is unclear, removal of the Egr1 gene from β1-integrin conditional knockout lenses partially rescued their fibrotic/apoptotic phenotype [61]. Thus, we investigated the possible function of Egr1 in the LEC injury response using Egr1 knockout (Egr1KO) mice [29].

We first confirmed that Egr1KO mice did not express the Egr1 protein (and validated the Egr1 antibody used in this study) by quantitative immunofluorescence (Supplementary Figure S1). Morphologically, adult Egr1KO lenses were the same size as those from their wildtype littermates, but exhibited subtle refractive abnormalities and focal opacities. Hematoxylin and eosin (H&E) staining suggest that Egr1KO lenses are phenotypically similar to wildtype although they may have a slight disorganization of their transition zone (Supplementary Figure S1) suggesting that Egr1 may play a role in regulating adult lens structure.

### 3.5. Egr1 May Mediate a Portion of the Injury Response of LECs PCS

**Figure 6:**
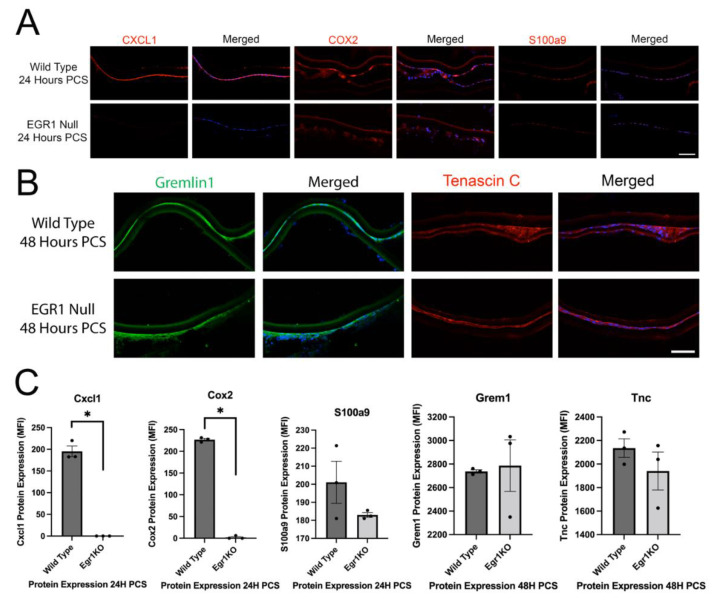
Egr1KO LECs attenuate a portion of their inflammatory response PCS but the fibrotic response was not affected. (**A**) Immunofluorescence analysis of the proinflammatory mediators Cxcl1, Cox2 and S100a9 (red) in LECs from wild type and Egr1KO mice at 24 h PCS. Blue = DNA, Scale bar = 70 μm. (**B**) Immunofluorescence analysis of the fibrotic markers Gremlin1 (green) and Tenascin C (red) in LECs from wild type and Egr1KO mice at 48 h PCS. Blue = DNA, Scale bar = 70 μm. (**C**) Quantitation of the data shown in panels A and B. Cxcl1 and Cox2, * represents *p* ≤ 0.01; S100a9, *p* = 0.26; Gremlin 1, *p* = 0.84; Tenascin C, *p* = 0.42. The black dots in C represent the individual biological replicates for each bar in the bar graph.

To investigate the role of Egr1 on the inflammatory and fibrotic response of LECs PCS, we first conducted a candidate gene approach. As previously described [18], we found that Cxcl1, Cox2 and S100a9 protein expression are all upregulated in wild type LECs at 24 h PCS while the upregulation of Cxcl1 and Cox2 was attenuated in Egr1KO mice. However, it appears that the inflammatory response is not globally attenuated as S100a9 expression upregulated normally in Egr1KO LECs at 24 h PCS (Figure 6A,C). Further protein levels for the fibrotic markers Gremlin1 and Tenascin C upregulate in wildtype LECs by 48 h PCS as previously reported [11,62] and this upregulation was not affected in Egr1KO LECs (Figure 6B,C) even though Egr1 has been reported to regulate the fibrotic response in other systems [63].

### 3.6. Deletion of the IETF Egr1 Only Slightly Affects the Transcriptome of Uninjured LECs but Altered a Portion of the Acute LEC Injury Response

As the function of Egr1 in the adult lens was still obscure following phenotypic and candidate gene investigations, RNAseq was then performed on wildtype and Egr1KO LECs isolated either immediately after lens fiber cell removal surgery or 6 h PCS, a time when Egr1 levels are robustly elevated at both the mRNA and protein levels (Figure 3 and Figure 4).

Comparison of wild type and Egr1KO LECs isolated immediately after lens fiber cell removal revealed that only 59 genes were differentially expressed by at least two-fold (FDR corrected *p* value ≤ 0.05), with 13 of these genes being upregulated and 46 being downregulated in Egr1KO LECs. Advaita iPathway guide analysis of the DEGs did not reveal any significantly impacted pathways common between the genes, although, 28 of the DEGs did map to the gene ontology term “cellular response to stimulus” (FDR corrected *p* = 0.04). These include the reduced expression of several genes encoding known regulators or targets of the MAPK pathway including Dusp5, Dusp10, Socs3, Arc, Ier5, Mt2 in naïve Egr1KO LECs (Supplementary Table S4; data deposited in the gene expression omnibus under #GSE206574).

Comparison between the transcriptomes of Egr1KO and wildtype LECs at 6 h PCS revealed 115 genes to be significantly differentially expressed by at least two fold (FDR corrected *p* value ≤ 0.05), with 72 of these genes being upregulated and 43 being downregulated DEGs. Filtering these DEGs for expression changes likely to be biologically significant [44], revealed 73 DEGs with 52 DEGs being upregulated and 21 DEGs downregulated in Egr1KO LECs at 6 h PCS compared to wild type (Supplementary Table S5/GEO#GSE206574). The most upregulated and downregulated genes in Egr1KO LECs at 6 h PCS can be found in Table 4 and Table 5, respectively.

### 3.7. Egr1KO LECs Upregulate Zonule Associated Genes upon Lens Injury

Inspection of the DEGs in Egr1KO LECs at 6 h PCS revealed that the mRNA levels of fibrillin 1 and fibrillin 2, two components of the ciliary zonule that maintains the position of the lens within the eye [64,65], were upregulated compared to wild type LECs at 6 h PCS. In order to evaluate Egr1’s involvement in zonular homeostasis, we compared the set of genes differentially expressed between wildtype and Egr1KO LECs to a set of 91 human proteins that proteomic analysis indicates to be the primary constituents of zonular fibers [65]. We observed 5 mouse homologs of human zonular genes with biologically significant and elevated differential expression at six hours PCS in Egr1KO LECs compared to WT (Table 6). This suggests that acute elevation of Egr1 levels apparently represses injury-associated elevation of zonule gene expression in LECs.

**Figure 7:**
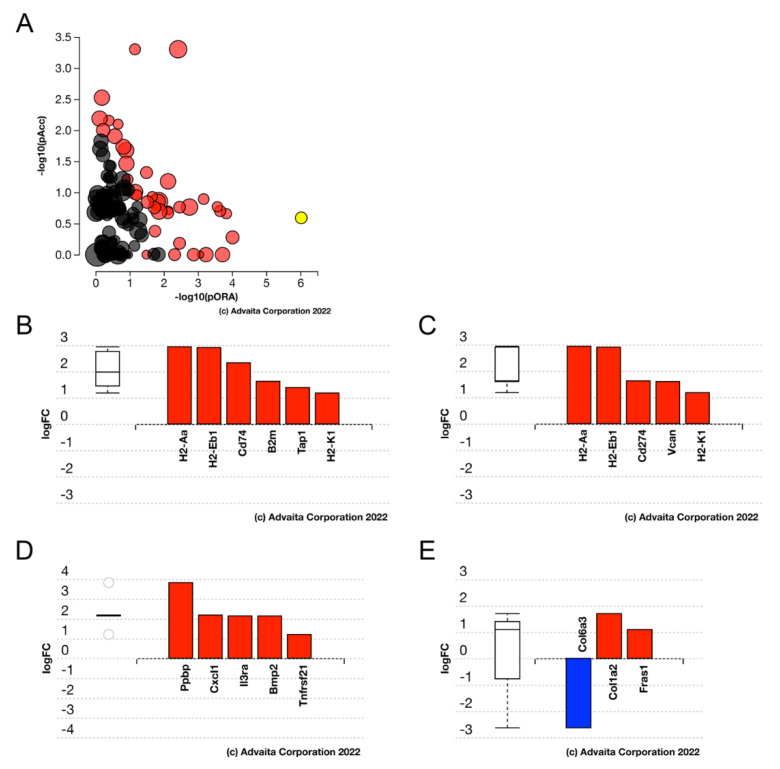
Advaita iPathway analysis of differentially expressed pathways/genes between 6 h Egr1KO and 6 h WT LECs. (**A**) Impact analysis of the DEGs suggest that the KEGG pathway map “antigen processing and presentation” (*p*-value = 4.147 × 10^−6^) (yellow dot) is the most significantly impacted pathway in the 6 h LECs. Other impacted KEGG pathways are represented by the other dots with black dots being non-significant KEGG pathways and red dots being significant KEGG pathways. (**B**) Bar graph showing the antigen processing and presentation genes that are differentially expressed in the 6H Egr1KO LECs. (**C**) Bar graph showing the cell adhesion molecules (*p*-value = 0.003) pathway of genes differentially expressed in the 6H Egr1KO LECs. (**D**) Bar graph showing the cytokine-cytokine receptor interaction (*p*-value = 0.003) pathway of genes differentially expressed in the 6H Egr1KO LECs. (**E**) Bar graph showing the ECM receptor interaction (*p*-value = 0.045) pathway of genes differentially expressed in the 6H Egr1KO LECs.

Advaita iPathway analysis of the DEGs in Egr1KO LECs at 6H revealed that the “antigen processing and presentation” pathway (*p*-value = 4.147 × 10^−6^) was the pathway most impacted by Egr1 gene deletion. In addition, genes mapping to the “cytokine-cytokine receptor interaction” (*p*-value = 0.003), “cell adhesion molecule” (*p*-value = 0.003) and ECM receptor interaction (*p*-value = 0.045) gene ontology terms were enriched in the Egr1KO 6H DEGs (Figure 7).

### 3.8. Generation, Validation and Morphological Analysis of FosB Conditional Knock Out Mice

**Figure 8:**
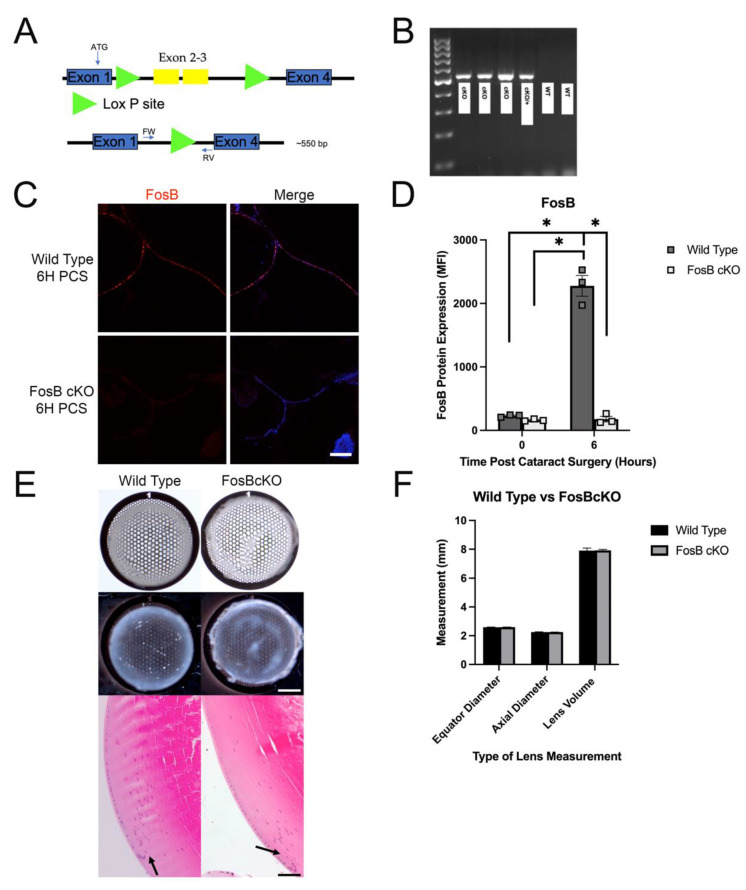
Creation of mice lacking the FosB gene from the lens (FosBcKO) and characterization of its lens phenotype. (**A**) Diagram of the floxed FosB allele before and after cre recombinase mediated deletion. The FosB allele used in this study is flanked by 2 Lox *p* sites (green triangles) around exons 2 and 3. After recombination, the FosB allele has exons 2 and 3 excised. Location of the genotyping primers used to confirm successful excision is indicated by FW and RV. (**B**) PCR analysis of lens DNA obtained from FosBcKO mice showing successful removal of exons 2 and 3. (**C**) Protein validation of the absence of the FosB protein (Red) in FosBcKO LECs via immunofluorescence at 6H PCS (Scale bar 70 µm) (Blue is DNA). (**D**) Quantification of the protein validation experiment in (**C**) * *p*-value < 0.0001. (**E**) Grid analysis, dark field imaging (Scale bar 50 µm) and H&E staining of the FosBcKO lens (right images) compared to the Wild type lens (left images) (Scale bar 100 µm). The black arrows are utilized to point out the disorganization in the transition zone of the lens. (**F**) Quantification of lens measurements obtained from the FosBcKO lens showing that they were of similar size to wildtype.

After the investigation of the Egr1KO LEC injury phenotype, the role of IETFs in regulating the response of LECs to lens fiber cell removal was still obscure. Thus, we investigated the role of FosB in the acute response of LECs to lens injury as it was one of the most upregulated genes (Table 1) and most upregulated IETFs (Table 3) at 6 h PCS. As FosB null mice exhibit nurturing defects [66], we generated mice lacking a functional FosB gene just in the lens (FosBcKO) by breeding a mouse carrying a floxed FosB allele [30] to a mouse containing the MLR10 lens specific cre recombinase transgene [31] (Figure 8A). The complete deletion of the floxed region of the FosB gene was validated by PCR analysis of DNA from mouse lens (Figure 8B) and further confirmation was validated at the protein level via immunofluorescence (Figure 8C,D). FosBcKO lens were the same size as the WT lens (Figure 8F), but similar to the Egr1KO lens, exhibit focal opacities visible by both brightfield and darkfield imaging (Figure 8E). Hematoxylin and eosin (H&E) staining demonstrated that FosBcKO lenses and WT lenses are histologically similar but FosBcKO appear to have a slight disorganization of the transition zone (Figure 8E).

### 3.9. Acute FosB Elevation PCS May Regulate a Portion of Both the Inflammatory and Fibrotic Responses Post Cataract Surgery

**Figure 9:**
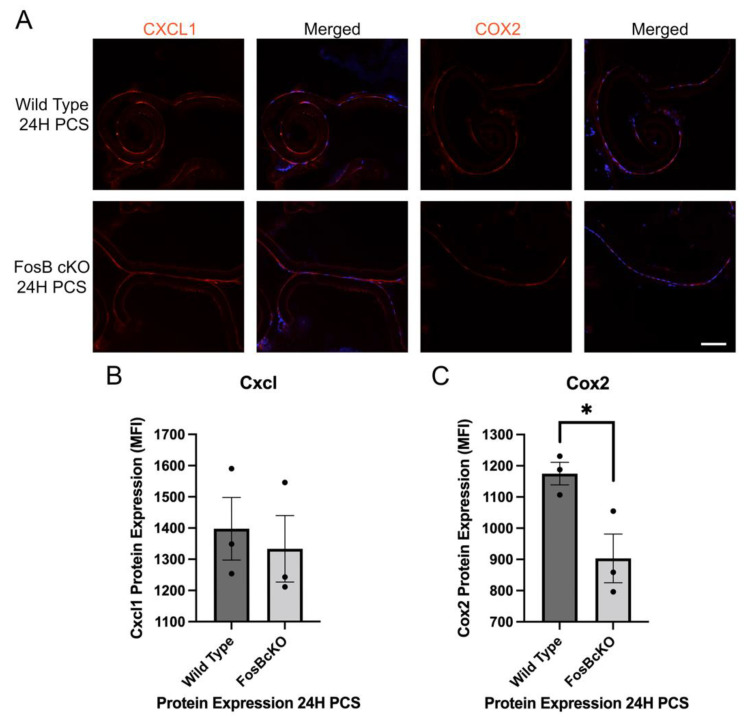
Inflammatory mediator protein expression in FosBcKO LECs compared to Wild Type at 24 h PCS. (**A**) Immunofluorescent staining for the proinflammatory mediators Cxcl1 and Cox2 (red) at 24 h PCS. Blue is DNA and Scale bar = 70 µm. (**B**) Quantification of Cxcl1 levels in LECs at 24 h PCS by immunofluorescence *p* = 0.97. (**C**) Quantification of Cox2 levels in LECs at 24 H PCS by immunofluorescence * represents *p*-value ≤ 0.01. The black dots in B & C represent the individual biological replicates for each bar in the bar graph.

**Figure 10:**
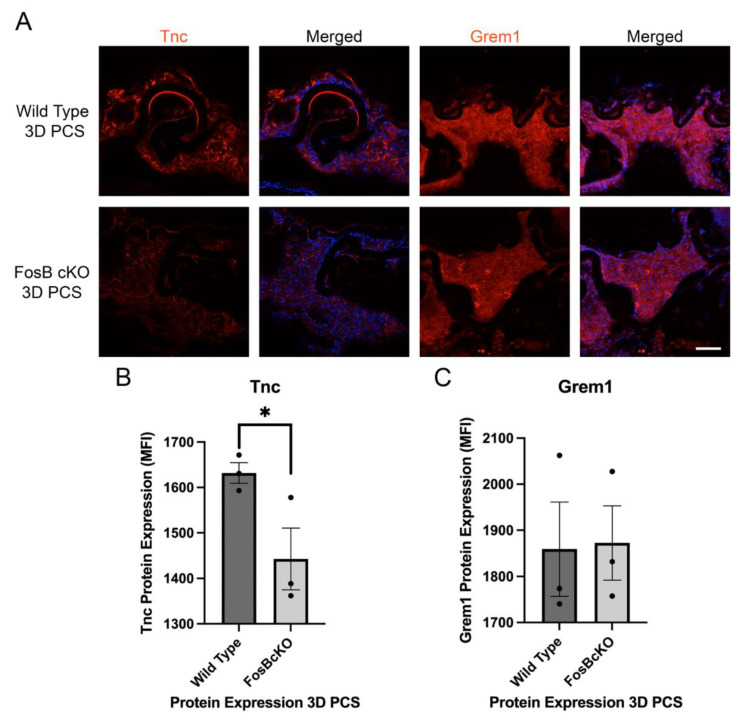
Fibrotic marker protein expression in FosBcKO LECs at 72 h PCS via immunofluorescence. (**A**) The fibrotic markers, Gremlin1 and Tenascin C (red) in wild type and and FosBcKO LECs at 72 h PCS. Blue = DNA, Scale bar = 70 μm. (**B**) Quantitation of Tenascin C protein levels in LECs at 72 h PCS analyzed by immunofluorescence * *p*-value ≤ 0.05. (**C**) Quantitation of Gremlin1 protein levels in LECs at 72 h PCS analyzed by immunofluorescence *p* = 0.99. The black dots in B & C represent the individual biological replicates for each bar in the bar graph.

To investigate the role of FosB on the inflammatory response PCS, we first conducted a candidate gene approach based upon the top upregulated and expressed inflammatory cytokines found in [18]. This analysis revealed that Cox2 expression was significantly downregulated in FosBcKO mice at 24 h PCS although Cxcl1 levels were not significantly affected (Figure 9). At 72 h PCS, a time point where fibrotic markers upregulate robustly in injured LECs, FosBcKO LECs exhibit much less Tenascin C upregulation than normal although levels of the fibrotic marker of Gremlin1 was similar between FosBcKO and wild type LECs (Figure 10).

### 3.10. Acute FosB Upregulation PCS Regulates Many Genes in Injured LECs

To further investigate the role of FosB in naïve LECs and the LEC injury response, RNAseq was conducted on LECs isolated from wild type and FosBcKO mice at 0 h and 6 h PCS. Notably, uninjured FosBcKO LECs only expressed 43 genes at levels statistically different from wild type, only 25 of which met the biological significant criteria of [44]) showing that the naïve lens transcriptome is only minimally impacted by the absence of FosB (Supplementary Table S6/GEO#GSE206574). However, comparison between FosBcKO and wildtype LECs at 6 h PCS revealed 874 genes to be differentially expressed, 589 meeting the biological significance criteria of [44], 485 DEGs being upregulated and 104 downregulated in FosBcKO LECs (Supplementary Table S7/GEO#GSE206574). The top most upregulated and downregulated genes in this comparison are presented in Table 7 and Table 8.

Several DEGs at 6 h PCS with higher expression in FosBcKO LECs compared to wildtype appeared to be associated with zonule formation. As a result, a list of zonules genes from a previous study [65] were used as a filter and revealed that FosBcKO LECs upregulate the expression of zonule genes upon lens injury and at 6 h PCS, this resulted in their elevated expression compared to wildtype (Table 9).

**Figure 11:**
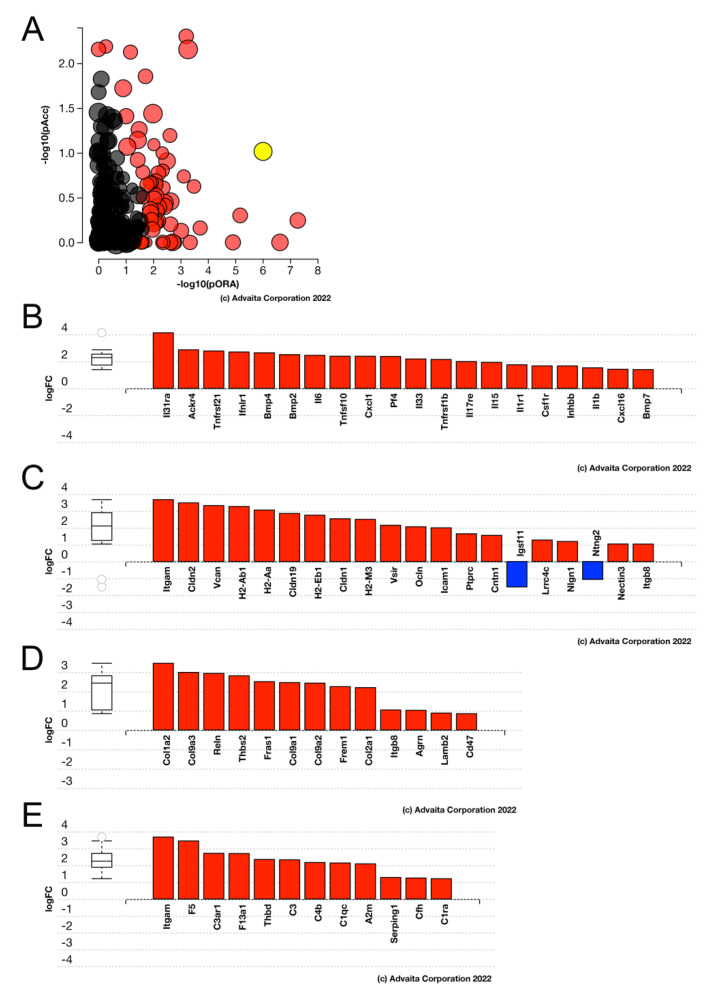
Advaita iPathway analysis of differentially expressed pathways/genes between 6 h FosBcKO and 6 h WT LECs. (**A**) Impact analysis of the DEGs suggest that the KEGG pathway map “cytokine-cytokine receptor interaction” (*p*-value = 1.655 × 10^−6^) (yellow dot) is the most significantly impacted pathway in the 6 h LECs. Other impacted KEGG pathways are represented by the other dots with black dots being non-significant KEGG pathways and red dots being significant KEGG pathways. (**B**) Bar graph showing the cytokine-cytokine receptor interaction genes that are differentially expressed in the 6 h FosBcKO LECs. (**C**) Bar graph showing the cell adhesion molecules (*p*-value = 7.754 × 10^−6^) pathway of genes differentially expressed in the 6 h FosBcKO LECs. (**D**) Bar graph showing the ECM-receptor interaction (*p*-value = 4.252 × 10^−5^) pathway of genes differentially expressed in the 6 h FosBcKO LECs. (**E**) Bar graph showing the complement and coagulation cascades (*p*-value = 4.528 × 10^−5^) pathway of genes differentially expressed in the 6 h FosBcKO LECs.

Advaita iPathway analysis revealed that the lack of FosB in LECs compared to WT LECs causes the “cytokine-cytokine receptor interaction” pathway (*p*-value = 1.655 × 10^−6^) to be the most upregulated signaling pathway (Figure 11). In addition, there is an upregulation of cell adhesion molecules (*p*-value = 7.754 × 10^−6^), ECM-receptor interaction (*p*-value = 4.252 × 10^−5^), and complement and coagulation cascades (*p*-value = 4.528 × 10^−5^) when there is the absence of FosB in the LECs PCS compared to WT LECs PCS.

## 4. Discussion

Extracapsular cataract extraction followed by intraocular lens implantation [3] is a marvel of modern medicine that has greatly reduced the worldwide burden of cataract-associated visual disability. While great methodological advances have improved the outcomes of this procedure over the past 50 years, post-surgical ocular inflammation [6] can still be problematic in the short term. Longer term, patients still have a significant risk of developing posterior capsular opacification (PCO) during the first decade following surgery [24]. Inflammation following cataract surgery is usually attributed to surgically induced breaks in the blood-aqueous barrier [25,26], and PCO is attributed to elevated TGFβ signaling [12,67] in lens epithelium cells retained on the lens capsular bag following surgery. However, the mechanisms by which cataract surgery induces these negative sequelae are still obscure.

Our prior work found that inflammation markers upregulate in LECs following lens fiber cell removal as early as 6 h post cataract surgery (PCS) with maximal upregulation by 24 h PCS [18] while it takes 48–72 h after surgery for TGFβ signaling leading to lens epithelial cell (LEC) fibrotic responses to elevate [18,36,62]. Here, we used RNAseq to globally evaluate the changes in LEC biology at 6 h PCS and identified a group of transcription factors that might be regulating the LEC injury response which contributes to ocular inflammation and PCO.

### 4.1. LECs Rapidly Induce the Expression of Pro-Inflammatory Cytokines after Lens Fiber Cell Removal but Fibrotic Responses Are More Delayed

Here, RNAseq confirmed that LECs upregulate the expression of numerous genes encoding pro-inflammatory cytokines by 6 h PCS, including Il19 [68], Csf3 [69], Il6 [70], and Ptgs2 [71], consistent with our prior study showing that select proinflammatory cytokines upregulate at the protein level by 6 h PCS [18]. As signaling from such cytokines can both loosen endothelial tight junctions and chemoattract neutrophils and macrophages to sites of tissue injury [72,73,74,75], this suggests that the LECs left on the lens capsular bag following cataract surgery may contribute to the loosening of the blood-aqueous barrier that leads to ocular inflammation (flare plus cells) [16] that is apparent by 24 h PCS.

Further, while remnant LECs still express most lens epithelial cell markers at pre-injury levels at 6 h PCS and did not upregulate mRNAs encoding classic fibrotic markers by this time, they already appear to have elevated the expression of numerous genes needed to set the stage for PCO pathogenesis. Particularly, the upregulated DEGs in LECs at 6 h PCS are enriched in those known to participate in epithelial-mesenchymal transition (EMT) and cell migration pathways which are important for PCO pathogenesis [76]. Notably, these genes are induced in LECs 1–2 days prior to upregulation of the αVβ8-integrin [11] expression needed to activate latent TGFβ and subsequent Smad2/3 phosphorylation which drives the long-term fibrotic response of LECs PCS. These data suggest that LECs are rapidly reprogrammed in response to lens injury/cataract surgery and this sets the stage for later TGFβ pathway mediated loss of lens epithelial marker gene expression and induction of the myofibroblast phenotype.

### 4.2. Mitogen Activated Protein Kinase Signaling and Immediate Early Transcription Factor Expression Are Rapidly Induced in LECs |Following| Lens Fiber Cell Removal

Bioinformatic analysis of the DEGs detected in LECs at 6 h PCS found that many genes map to the gene ontology term, mitogen activated protein kinase (MAPK) signaling. Further, direct inspection of the DEGs found that the expression of numerous immediate early transcription factors (IETFs), which are known to be the first genes whose expression is elevated by MAPK signaling in other systems [77,78], were elevated by 6 h PCS. We subsequently experimentally confirmed that these observations which suggests that the lens wound healing response is triggered by an event occurring during cataract surgery that leads to acute MAPK pathway activation and subsequent IETF expression. As isolated LEC/lens capsule complexes subjected to 6 h of ex vivo serum-free culture also induce the expression of IETFs, this response appears to be autonomous to LECs themselves.

While future work is needed to identify the trigger of MAPK signaling, there are several possibilities. Danger-associated molecular patterns (DAMPs) are intracellular proteins which are released upon cellular injury. They can bind to DAMP receptors (Toll-like receptors are the best characterized members of this class) which activates several downstream signaling pathways including MAPK [79]. While cataract surgery would be expected to release known DAMPs such as αA- and αB- crystallins [80] into the ocular environment, inspection of the LEC transcriptome for DAMP receptors suggested that this pathway may not be the acute trigger of the LEC injury response as their expression levels are low in naïve LECs. However, DAMP receptors may play a role in later LEC responses post-surgery as their expression elevates at later times PCS. Alternatively, the biomechanical stress placed on the remnant LECs during fiber cell removal could be the trigger. Biomechanical stress on the lens has been reported to acutely induce the expression of IETFs in LECs [81,82] and when the biomechanical stress is high or prolonged, anterior subcapsular cataract develops [83,84], which like PCO, often involves the EMT of LECs [85]. Notably, naïve LECs do express some known biomechanical sensors including β1-integrin [61] and Piezo1 [86] which, when activated, upregulate the ERK/MAPK signaling cascade and subsequent IETF expression in other systems [87,88].

### 4.3. The IETFs Egr1 and FosB Regulate Some Aspects of the Lens Wound Healing Response

IETFs are known to regulate wound healing responses in non-lens cell types [89,90,91,92], however their functions in the lens are obscure. Here, we found that numerous IETFs upregulate rapidly in injured LECs then downregulate their expression later, although the kinetics of this downregulation varies between IETFs. Here, we tested the function of two of the most upregulated IETFs in LECs, Egr1 and FosB in the lens wound healing response as both genes can be under the control of ERK/MAPK signaling [77,78] and are known to regulate both inflammation [93,94] and fibrosis [63,89] in other tissues.

The absence of Egr1 does not induce major changes to normal lens biology either at the level of structure/transparency nor the global LEC transcriptome. This is consistent with prior reports identifying a role for Egr1 in scleral growth during compensatory myopia in mice although no effects on lens transparency are noted. Prior work has found that Egr1 upregulates in LEC in response to cellular stresses ranging from a loss of calcium homoeostasis due to selenite stress [59] and the loss of β1-integrin [61] while this study found Egr1 expression to rapidly elevate after lens fiber cell removal. However, the function of Egr1 in lens biology is still obscure. While we previously found that deletion of Egr1 from lenses lacking the gene encoding β1-integrin rescued [61] a portion of the LEC phenotype, the genes that Egr1 regulates that lead to lens destruction are still unknown. Here, we found that deletion of Egr1 from lenses subjected to injury only affected the expression of 73 genes, and surprisingly the handful of inflammatory cytokines among this list exhibited elevated expression in Egr1 null LECs at 6 h PCS, including Cxcl1 (4.55 fold), a known Egr1 target gene [94]. However, by 24 h PCS, Egr1 null LECs did exhibit reduced levels of Cxcl1 compared to wild type controls. While the mechanisms that underlay these results are unclear, Egr1 is known to function as both a transcriptional activator and repressor depending on context. LECs do express appreciable levels of the mRNAs encoding the Egr1 co-repressors Nab1 and Nab2 which could explain why Cxcl1 mRNA levels are elevated in Egr1 nulls at 6 h PCS as Cxcl1 is a known Egr1 target gene. It is possible that Egr1 shifts function later in the injury response as Cxcl1 levels are reduced at the protein level in Egr1 null LECs at 24 h PCS which is more consistent with other studies that suggest that Egr1 can activate the inflammatory response in non-lens cells [93,94]. It is also possible that the small effect of Egr1 deletion on the acute injury transcriptome derives from the large number of IETFs (who have similar or identical DNA binding sites) with elevated expression at 6 h PCS creating a “bulk” effect to drive the injury response so that loss of any one component only has a minor effect on the global injury response [95,96,97].

Like Egr1KO lenses, FosBcKO lenses exhibit neither major structural defects nor large changes in the LEC transcriptome. While it has been reported that elevated expression of the delta FosB (ΔFosB) variant in the lens can result in posterior subcapsular cataract [98], the apparently normal phenotype of FosBcKO lenses is not surprising in light of the low level of FosB expression in healthy LECs. In contrast, LECs lacking FosB exhibit many changes to their acute transcriptomic response to lens fiber cell removal with 874 DEGs detected in FosBcKO LECs at 6 h PCS. However, in contrast to our initial predictions, FosBcKO LECs exhibit higher expression of inflammatory markers than wildtype LECs at 6 h PCS.

FosB is a member of the AP1 family of transcription factors that mediates its function as either a homodimer or a heterodimer with other members of this family. The complexity of FosB function is further expanded by its numerous splice forms including ΔFosB which is a stable transcriptional repressor [99,100]. As the vast majority of the injury related DEGs detected in FosBcKO LECs are upregulated, including other IETFs such as JunB and Ier3 as well as Itgb8 (2 fold) which is critical for TGFβ activation [11] by injured LECs, it is possible that FosB elevation upon lens injury buffers the acute injury response by repressing the action of other IETFs.

## 5. Conclusions

This study revealed that lens epithelial cells that remain attached to the lens capsule following lens fiber cell removal modeling cataract surgery greatly reprogram their transcriptome by 6 h post surgery which likely sets the stage for subsequent post surgical ocular inflammation and fibrosis/PCO pathogenesis. The rapid elevation of MAPK signaling as measured by ERK phosphorylation post cataract surgery and the subsequent induction of immediate early transcription factor expression suggests that injury induced cell signaling driving the immediate early response sets the stage for the conditions needed to induce the epithelial-mesenchymal transition of lens epithelial cells to myofibroblasts. Further work is necessary to identify the molecular mechanisms by which lens fiber cell removal triggers this response and which IETFs play the most critical roles in setting the stage for later epithelial mesenchymal transition of lens epithelial cells and subsequent fibrotic PCO.

## Figures and Tables

**Table 1:** Top 20 most upregulated genes in mouse LECs at 6 h PCS.

Gene Symbol	Gene Name	Wild Type 0H FPKM	Wild Type 6H FPKM	Fold Change	FDR
Il19	Interleukin 19	0.00	3.38	753.97	4.39 × 10^−3^
Krtap4-16	Keratin associated protein 4–16	0.00	2.14	427.68	3.14 × 10^−3^
Csf3	Colony Stimulating Factor 3	0.11	21.34	232.91	1.31 × 10^−2^
Il6	Interleukin 6	0.07	8.55	149.03	4.61 × 10^−2^
Fosb	FBJ osteosarcoma oncogene B	1.89	198.30	130.49	9.36 × 10^−3^
Ptx3	Pentraxin 3	1.08	105.47	120.93	5.05 × 10^−3^
Esm1	Endothelial cell-specific molecule 1	0.16	12.52	96.90	4.56 × 10^−3^
Iqcn	IQ motif containing N	0.16	8.66	67.98	1.58 × 10^−2^
C2cd4b	C2 calcium-dependent domain containing 4B	0.15	8.17	65.46	8.63 × 10^−3^
Emp1	Epithelial membrane protein 1	1.85	76.36	51.28	4.39 × 10^−5^
Ifi202b	Interferon activated gene 202B	0.09	3.64	47.32	9.48 × 10^−3^
Arc	Activity regulated cytoskeletal associated protein	5.64	183.82	39.94	3.64 × 10^−3^
Ptgs2	Prostaglandin-endoperoxide synthase 2	0.65	20.96	39.62	7.93 × 10^−3^
Plaur	Plasminogen activator, urokinase receptor	0.18	5.54	36.69	4.17 × 10^−2^
Klf2	Kruppel-like factor 2	1.14	28.17	30.60	1.11 × 10^−3^
Htr1d	5-hydroxytrptamine receptor 1D	0.54	13.06	29.37	2.39 × 10^−5^
Rnf125	Ring finger protein 125	0.18	4.17	27.65	1.60 × 10^−3^
F3	Coagulation factor III	1.97	43.91	27.01	3.80 × 10^−3^
Lif	Leukemia inhibitory factor	1.50	33.10	26.40	1.41 × 10^−3^
Fgl2	Fibrinogen-like protein 2	0.56	11.78	26.39	3.12 × 10^−2^

**Table 2:** The expression of classic fibrotic markers does not significantly change in mouse LECs by 6 h PCS.

Gene Symbol	Gene Name	Wild Type 0 h FPKM	Wild Type 6 h FPKM	Fold Change	FDR
Col1a1	Collagen, type I, alpha 1	1.53	7.24	5.80	2.14 × 10^−1^
Tnc	Tenascin C	0.65	1.10	2.10	3.43 × 10^−1^
Acta2	α Smooth Muscle Actin	53.17	63.90	1.48	7.06 × 10^−1^
Itgav	Integrin alpha V	27.55	31.57	1.42	7.71 × 10^−2^
Vtn	Vitronectin	23.57	27.60	1.41	6.02 × 10^−1^
Fn1	Fibronectin 1	2.79	2.16	−1.03	9.70 × 10^−1^
Itgb1	Integrin beta 1	56.79	56.59	1.24	4.17 × 10^−1^

**Table 3:** IETFs differentially expressed in wildtype mouse LECs at 6 h PCS.

Gene Symbol	Gene Name	Wild Type 0 h FPKM	Wild Type 6 h FPKM	Fold Change	FDR
Fosb	FBJ osteosarcoma oncogene B	1.89	198.30	130.49	9.36 × 10^−3^
Klf2	Kruppel-like factor 2	1.14	28.17	30.60	1.11 × 10^−3^
Nr4a1	Nuclear receptor subfamily 4, group A, member 1	8.67	105.28	15.14	3.35 × 10^−2^
Zfp36	Zinc finger protein 36	13.93	163.10	14.56	7.59 × 10^−3^
Ier2	Immediate early response 2	10.16	111.54	13.66	1.43 × 10^−2^
Egr3	Early growth response 3	1.26	13.40	13.09	2.40 × 10^−2^
Ier5	Immediate early response 5	5.55	55.82	12.30	5.95 × 10^−5^
Egr1	Early growth response 1	33.41	328.89	12.10	3.09 × 10^−2^
Atf3	Activating transcription factor 3	33.02	268.96	10.17	3.76 × 10^−2^
Ier3	Immediate early response 3	3.45	28.17	9.94	2.35 × 10^−4^
Junb	Jun B proto-oncogene	20.98	160.07	9.51	2.97 × 10^−3^
Maff	V-maf musculoaponeurotic fibrosarcoma oncogene family, protein F	12.55	89.64	8.82	1.31 × 10^−4^
Klf4	Kruppel-like factor 4	25.06	166.32	8.21	3.38 × 10^−3^
Egr2	Early growth response 2	0.88	5.49	7.61	4.84 × 10^−2^

**Table 4:** Topmost upregulated DEGS in Egr1KO LECs at 6 h PCS.

Gene Symbol	Gene Name	Wild Type 6 h FPKM	Egr1KO 6 h FPKM	Fold Change	FDR
Pebp4	Phosphatidylethanolamine binding protein 4	0.14	2.18	14.58	3.33 × 10^−3^
Dio3	Deiodinase, iodothyronine type III	0.38	3.29	8.56	9.90 × 10^−4^
H2-Aa	Histocompatibility 2, class II antigen A, alpha	0.37	2.88	7.67	4.98 × 10^−3^
H2-Eb1	Histocompatibility 2, class II antigen E beta	0.87	6.63	7.50	1.23 × 10^−2^
Cd74	CD74 antigen	4.77	24.04	5.02	2.73 × 10^−2^
Cxcl1	Chemokine (C-X-C motif) ligand 1	15.11	68.71	4.55	7.51 × 10^−7^
Fbn2	Fibrillin 2	0.60	2.65	4.42	2.60 × 10^−3^
Il3ra	Interleukin 3 receptor, alpha chain	0.71	3.17	4.41	2.79 × 10^−2^
Bmp2	Bone morphogenetic protein 2	5.37	23.64	4.40	5.02 × 10^−4^
Slc6a13	Solute carrier family 6, member 13	2.60	11.31	4.34	3.62 × 10^−3^

**Table 5:** Topmost downregulated DEGs seen in Egr1KO LECs at 6 h PCS.

Gene Code	Gene Name	Wild Type 6 h FPKM	Egr1KO 6 h FPKM	Fold Change	FDR
Sst	Somatostatin	6.71	0.13	−39.55	4.98 × 10^−3^
Krt5	Keratin 5	14.06	0.64	−21.81	2.89 × 10^−2^
C1ql2	Complement component 1	4.94	0.68	−7.08	9.20 × 10^−3^
Rit2	Ras-like without CAAX 2	3.51	0.52	−6.66	4.24 × 10^−2^
Zfp804a	Zinc finger protein 804A	2.42	0.37	−6.32	2.22 × 10^−2^
Col6a3	Collagen, type VI, alpha 3	36.99	5.97	−6.19	3.91 × 10^−3^
Spc25	SPC25	14.50	2.88	−5.02	2.14 × 10^−3^
Map2k3os	Mitogen-activated protein kinase kinase 3, opposite strand	4.04	0.77	−5.01	2.89 × 10^−2^
Rab7b	RAB7B, member Ras oncogene family	4.88	1.01	−4.79	2.22 × 10^−2^
Galnt13	Polypeptide N-acetylgalactosaminyltransferase13	2.76	0.64	−4.25	2.22 × 10^−2^

**Table 6:** Egr1KO LECs express elevated levels of genes that encode zonule associated proteins at 6 h PCS.

Gene Code.	Gene Name	Wild Type 6 h FPKM	Egr1KO 6 h FPKM	Fold Change	FDR
Fbn2	Fibrillin 2	0.60	2.65	4.42	2.60 × 10^−3^
Fbn1	Fibrillin 1	8.27	30.34	3.67	1.55 × 10^−2^
Col18a1	Collagen, type XVIII, alpha 1	21.09	71.66	3.40	2.79 × 10^−2^
Ltbp2	Latent transforming growth factor beta binding protein 2	6.54	21.56	3.30	2.70 × 10^−2^
Ltbp1	Latent transforming growth factor beta binding protein 1	6.54	17.41	2.66	5.22 × 10^−3^

**Table 7:** Top most upregulated DEGs in FosBcKO LECs at 6 h PCS.

Gene Code	Gene Name	Wild Type 6 h FPKM	FosBcKO 6 h FPKM	Fold Change	FDR
Dio3	Deiodinase, iodothyronine type III	0.39	10.75	27.00	4.19 × 10^−24^
Pebp4	Phosphatidylethanolamine binding protein 4	0.14	4.11	26.88	4.75 × 10^−12^
Clec14a	C-type lectin domain family 14, member a	0.09	2.48	24.75	2.60 × 10^−8^
Edn3	Endothelin 3	0.22	3.76	17.00	1.78 × 10^−8^
Foxi3	Forkhead box i3	0.83	12.46	14.80	1.39 × 10^−10^
Kcnj13	Potassium inwardly rectifying channel, subfamily J, member 13	0.74	11.13	14.74	6.61 × 10^−8^
Defb9	Defensin beta 9	0.80	12.02	14.46	2.14 × 10^−7^
Fbn2	Fibrillin 2	0.61	8.81	14.27	3.60 × 10^−14^
Dsg2	Desmoglein 2	0.36	5.07	13.78	6.46 × 10^−24^
Gpx3	Glutathione peroxidase 3	55.77	735.90	13.19	1.38 × 10^−14^

**Table 8:** Top most downregulated DEGs in FosBcKO LECs at 6 h PCS.

Gene Code	Gene Name	Wild Type 6 h FPKM	FosBcKO 6 h FPKM	Fold Change	FDR
Slc29a4	Solute carrier family 29	6.89	0.87	−7.86	2.95 × 10^−4^
Sox30	SRY box 30	3.17	0.43	−7.24	1.17 × 10^−3^
Gjb3	Gap junction protein, beta 3	4.37	0.62	−7.06	2.73 × 10^−2^
Cdcp3	CUB domain containing protein 3	8.71	1.60	−5.43	1.54 × 10^−8^
Gjb4	Gap junction protein, beta 4	8.02	1.52	−5.29	3.36 × 10^−3^
H1f8	H1.8 linker histone	35.69	6.96	−5.13	1.18 × 10^−3^
Cdsn	Corneodesmosin	3.16	0.75	−4.21	1.12 × 10^−4^
Alpk2	Alpha-kinase 2	3.24	0.78	−4.16	5.48 × 10^−3^
Asb5	Ankyrin repeat and SOCs box-containing 5	6.02	1.45	−4.16	5.48 × 10^−3^
Cd109	CD109 antigen	4.25	1.06	−4.01	1.71 × 10^−2^

**Table 9:** Genes encoding zonule components that are upregulated in FosBcKO LECs at 6 h PCS compared to wildtype.

Gene Code	Gene Name	Wild Type 6 h FPKM	FosBcKO 6 h FPKM	Fold Change	FDR
Fbn2	Fibrillin 2	0.61	8.81	14.27	3.60 × 10^−14^
Fbn1	Fibrillin 1	8.52	105.46	12.38	6.38 × 10^−11^
Col18a1	Collagen, type XVIII, alpha 1	21.68	233.53	10.77	1.43 × 10^−9^
Ltbp2	Latent transforming growth factor beta binding protein 2	6.72	72.04	10.71	2.55 × 10^−10^
Nid2	Nidogen 2	1.59	16.90	10.58	4.08 × 10^−10^
Efemp1	Epidermal growth factor-containing fibulin-like extracellular matrix protein 1	18.34	135.34	7.37	4.46 × 10^−12^
Prelp	Proline arginine-rich end leucine-rich repeat	14.44	83.96	5.81	3.11 × 10^−11^
Col9a2	Collagen, type IX, alpha 2	6.41	34.69	5.41	1.78 × 10^−8^
Hmcn1	Hemicentin 1	0.95	4.20	4.41	1.09 × 10^−10^
Ltbp1	Latent transforming growth factor beta binding protein 1	6.75	24.79	3.67	1.32 × 10^−10^
Megf	Multiple EGF-like domains 6	1.27	4.39	3.45	4.86 × 10^−5^
Loxl1	Lysyl oxidase-like 1	26.12	64.47	2.47	6.21 × 10^−4^
Ltbp3	Latent transforming growth factor beta binding protein 3	23.90	52.78	2.21	1.58 × 10^−4^
Ctsd	Cathepsin D	31.84	70.16	2.20	4.60 × 10^−5^
Timp3	Tissue inhibitor of metalloproteinase 3	94.83	198.21	2.09	1.40 × 10^−2^
Tgfb3	Transforming growth factor, beta 3	2.83	5.74	2.03	1.21 × 10^−2^
Agrn	Agrin	8.37	16.96	2.03	1.42 × 10^−3^

## Data Availability

The RNAseq data presented in this manuscript can be obtained at the Gene Expression Omnibus under accession numbers GSE206563 and GSE206574.

## Supplementary Materials

The following supporting information can be downloaded at: https://www.mdpi.com/article/10.3390/cells11213456/s1, Figure S1: Phenotype of the adult Egr1KO lens; Table S1: List of all qRT-PCR primers; Table S2: Primary antibodies used in this study; Table S3: Differential expression of genes between naïve WT LECs and those isolated 6 h PCS; Table S4: Genes differentially expressed in naïve adult mouse LECs due to deletion of the Egr1 gene; Table S5: Genes differentially expressed in between WT adult mouse LECs and those lacking the Egr1 gene at 6 hours PCS; Table S6: Genes differentially expressed in naïve adult mouse LECs due to deletion of the Fosb gene; Table S7: Genes differentially expressed in between WT adult mouse LECs and those lacking the Fosb gene at 6 hours PCS.
