# Supplementary material for: The Immediate Early Response of Lens Epithelial Cells to Lens Injury
Source: Cells. 2022 Nov 1;11(21):3456. doi: 10.3390/cells11213456 (PMC9654717; doi:10.3390/cells11213456)
Supplement: Supplementary file 1 [file cells-11-03456-s001.zip › Supplemental Table S1.pdf]

Supplemental Table S1: List of all qRT-PCR primers

Gene	Forward Primer	Reverse Primer
EGR1	5'-AAC-CCT-ATG-AGC-ACC-TGA-CC-3'	5'-GAG-AAG-CGG-CCA-GTA-TAG-GT-3'
FosB	5'-TAG-CTC-ATC-ACC-CTC-CGC-CGA-GTC-T-3'	5'-AGG-GTG-GGT-TGC-ACG-AGC-CAC-TGA-AG-3'
GAPDH	5'-CCG-CAT-CTT-CTT-GTG-CAG-T-3'	5'-GAA-TTT-TGC-CGT-GAG-TGG-AGT-3'
