# Supplementary material for: The Immediate Early Response of Lens Epithelial Cells to Lens Injury
Source: Cells. 2022 Nov 1;11(21):3456. doi: 10.3390/cells11213456 (PMC9654717; doi:10.3390/cells11213456)
Supplement: Supplementary file 1 [file cells-11-03456-s001.zip › Supplemental Table S2.pdf]

Supplemental Table S2: Primary antibodies used in this study

Primary Antibody	Fixation	Blocking Buffer	Primary Antibody Conditions
EGR1 (4153, Cell Signaling)	4% PFA	2% BSA, 5% NGS, and 0.3% Triton X-100 in PBS	1:100; overnight at 4°C
FosB (MA5-15056, Invitrogen)	4% PFA	2% BSA, 5% NGS, and 0.3% Triton X-100 in PBS	1:100; overnight at 4°C
c-Fos (2250, Cell Signaling)	4% PFA	2% BSA, 5% NGS, and 0.3% Triton X-100 in PBS	1:100; overnight at 4°C
JunB (3753, Cell Signaling)	4% PFA	2% BSA, 5% NGS, and 0.3% Triton X-100 in PBS	1:100; overnight at 4°C
c-Jun (9165, Cell Signaling)	4% PFA	2% BSA, 5% NGS, and 0.3% Triton X-100 in PBS	1:100; overnight at 4°C
CXCL1 (12335-1-AP, Proteintech	1:1 Acetone-Methanol	2% BSA in PBS	1:100; overnight at 4°C
COX-2/PTSG2 (ab15191, Abcam)	1:1 Acetone-Methanol	2% BSA and 5% NGS in PBS	1:200; 1 hour at RT

S100a9 (ab203133, Abcam)	1:1 Acetone-Methanol	2% BSA and 5% NGS in PBS	1:100; overnight at 4°C
Tenascin C (T3413, Sigma-Aldrich)	4% PFA	2% BSA in PBS	1:200; overnight at 4°C
Gremlin-1 (PA5-13123, Invitrogen)	4% PFA	2% BSA and 5% NGS in PBS	1:200; overnight at 4°C
pERK (9101S, Cell Signaling)	4% PFA	2% BSA, 5% NGS, and 0.3% Triton X-100 in PBS	1:100; overnight at 4°C
